# Occurrence, Species Distribution, and Antimicrobial Resistance of *Listeria* spp. in Foods from Retail and Farm Outlets in North Carolina, USA

**DOI:** 10.3390/microorganisms14081769

**Published:** 2026-08-11

**Authors:** Akbar Bahrami, Shurrita Davis, Erute Magdalene Adongbede, Janak Raj Khatiwada, Rishipal Rastrapal Bansode, Leonard Lamont Williams

**Affiliations:** Center for Excellence in Post-Harvest Technologies, North Carolina A&T State University, North Carolina Research Campus, Kannapolis, NC 28081, USA; bahrami.akbar999@gmail.com (A.B.); sd017437@ncat.edu (S.D.); emadongbede@ncat.edu (E.M.A.); jrkhatiw@ncat.edu (J.R.K.); rbansode@ncat.edu (R.R.B.)

**Keywords:** *Listeria monocytogenes*, food processing, retail foods, multidrug resistance, One Health

## Abstract

Contamination of retail foods by *Listeria* species, compounded by emerging antimicrobial resistance (AMR), presents a critical public health challenge that demands continuous surveillance. This study evaluated prevalence, species distribution, and AMR profiles of *Listeria* spp. isolated from 861 retail food samples in North Carolina, USA, (2018 and 2020). Isolation and molecular characterization were achieved through selective culturing, multiplex PCR, and antimicrobial susceptibility profiling via disk diffusion. *Listeria* was detected in 8.48% (73/861) samples, with 68 isolates identified to species level. Non-pathogenic strains predominated, led by *L. welshimeri* (24.66% of isolates) and *L. innocua* (21.92%). Pathogenic species (*L. monocytogenes*; *L. ivanovii*) represented 17.81% of isolates and were recovered exclusively from raw commodities. Vegetables yielded the most positive samples overall, but raw milk showed the highest individual category prevalence. Processed foods remained largely uncontaminated. Unprocessed foods were 27 times more likely to harbor *Listeria* than processed alternatives (RR = 27.19; 95% CI: 3.84–192.51. *p* < 0.0001). Phenotypic resistance was most frequent to Penicillin G (33.82%). Multidrug resistance (MDR) was identified in 64.71% of isolates, encompassing 84.62% of pathogenic and 60.00% of non-pathogenic species. Raw agricultural commodities serve as critical reservoirs for resistant *Listeria*, emphasizing the need for integrated surveillance frameworks.

## 1. Introduction

Foodborne diseases remain a major public health challenge globally, with bacterial pathogens contributing substantially to morbidity, mortality and associated economic losses [1]. Among these pathogens, *Listeria* spp., are especially important because they persist across agricultural environments, food-processing facilities, and retail foods, creating recurring risks throughout the farm-to-retail continuum [2]. The unique physiological resilience of the genus *Listeria* complicates mitigation strategies across the farm-to-fork continuum. *Listeria* species are ubiquitous environmental saprophytes capable of surviving under extreme environmental stresses. Notably, these psychrotrophic bacteria can actively proliferate at refrigeration temperatures (4–10 °C), tolerate low pH ranges, and withstand high sodium chloride concentrations. These characteristics allow them to colonize and persist within food processing plant niches, creating persistent reservoirs that can lead to post-processing cross-contamination. While thermal processing effectively eliminates the pathogen, fresh, minimally processed, or post-harvest exposed foods remain highly susceptible to contamination [3]. Furthermore, *Listeria* spp. readily forms biofilms on food-contact surfaces, including stainless steel equipment, conveyor belts, drains, and packaging systems. Biofilm formation enhances persistence within the food-processing environments and contributes to recurring contamination events despite routine sanitation procedures [4].

The taxonomy of the genus *Listeria* has expanded over the past two decades with tens of novel species from environmental and agricultural habitats. However, six species—*L. monocytogenes*, *L. ivanovii*, *L. innocua*, *L. seeligeri*, *L. grayi* and *L. welshimeri*, remain of relevance in food microbiology because of their frequent association with food products and food processing environments. Among these, *L. monocytogenes* is the primary etiological agent in human listeriosis, whereas *L. ivanovii* is principally associated with veterinary infections (ruminants), although sporadic human infections have also been reported [5,6]. Other species are generally considered non-pathogenic but are frequently recovered from food and environmental sources, where they may indicate contamination pressure, hygiene status, and ecological conditions that support *Listeria* persistence [6].

*L. monocytogenes* remains the species of greatest clinical concern because it causes severe invasive disease and high mortality despite relatively low incidence. Temperature-dependent motility may contribute to the environmental fitness of L. monocytogenes, which is non-motile at 37 °C but highly motile at 22–28 °C [7]. Clinical manifestations range from mild gastroenteritis to severe systemic infections, including septicemia, meningitis, encephalitis, maternal–fetal infections, spontaneous abortion, stillbirth, and neonatal disease [8,9]. Mortality may exceed 20–30% among pregnant women, neonates, older adults, and immunocompromised individuals [7]. In the United States, *L. monocytogenes* remains a priority foodborne pathogen because of its disease burden and continued involvement in recalls and outbreaks [10,11]. It is also included by the Interagency Food Safety Analytics Collaboration among priority pathogens for source attribution and action, along with *Campylobacter* spp., nontyphoidal *Salmonella*, and Shiga toxin-producing *Escherichia coli* (STEC) [12].

Although *L. monocytogenes* drives most public health concern, non-pathogenic *Listeria* species are frequently recovered from the same foods and environmental niches [13]. Because these species may share adaptations that promote persistence, their detection can strengthen hygiene assessment, risk interpretation, and understanding of *Listeria* ecology and transmission within food systems [9,13].

*Listeria* contamination can occur at multiple points along the food supply chain. Fresh produce, edible fungi (mushrooms), and other raw agricultural commodities may be exposed through soil, irrigation water, manure, wildlife, or other environmental reservoirs [11], whereas meat and dairy products may be contaminated during production, slaughter, milking, processing, transport, or retail handling [14]. Although pasteurization, thermal processing, sanitation, and Hazard Analysis and Critical Control Point (HACCP) systems reduce microbial hazards, post-processing, contamination remains difficult to eliminate [12]. Surveillance across both raw and processed foods is therefore essential for identifying contamination routes and improving control strategies.

Reliable detection remains central to *Listeria* surveillance because contamination levels may be low and cells can be stressed or injured by processing. Regulatory protocols therefore rely on selective enrichment, plating, and biochemical confirmation [11]. Chromogenic media, including Agar *Listeria* according to Ottaviani and Agosti (ALOA), improve recovery and differentiation, while multiplex polymerase chain reaction (PCR) enables faster species-level identification for surveillance and epidemiological investigations [15,16].

In addition to contamination concerns, the global emergence of antimicrobial resistance (AMR) among *Listeria* species has become an increasing public health challenge [17]. Although listeriosis is traditionally treated with β-lactam antibiotics particularly ampicillin or penicillin, often in combination with aminoglycosides, growing reports of resistance to clinically important antimicrobial agents have raised concerns about long-term treatment efficacy [18,19].

Non-pathogenic *Listeria* species may also serve as environmental reservoirs of phenotypic AMR in food production systems, supporting genus-wide susceptibility monitoring rather than surveillance focused only on *L. monocytogenes* [2].

Effective control requires coordinated regulatory and industry interventions, including surveillance programs, microbiological standards, environmental monitoring, thermal and non-thermal processing, biological control agents such as nisin, and chemical preservatives. Because performance varies by food matrix, application method, and processing condition, these interventions require continued evaluation under commercial conditions. Despite extensive commodity-specific research, integrated data comparing *Listeria* species distribution, pathogenicity profiles, and AMR patterns across multiple retail food categories remain limited.

To address this gap this study evaluated the prevalence, species distribution, and phenotypic antimicrobial profiles of *Listeria* spp. isolated from diverse retail food commodities in North Carolina, USA. Using fifteen clinically and agriculturally relevant antimicrobials, the study assessed susceptibility patterns among pathogenic and environmental strains and generates data to support integrated food safety and One Health surveillance.

Consumer demand for fresh, raw, and ready-to-eat foods has shifted the epidemiology of foodborne listeriosis. Although earlier outbreaks were mainly linked to processed meats and soft cheeses, recent outbreaks increasingly involve fresh produce, including leafy greens, stone fruits, and melons, underscoring vulnerabilities in raw agricultural supply chains where post-harvest kill steps are limited. Environmental monitoring therefore often includes non-pathogenic indicators such as *L. innocua*, which shares survival niches and growth characteristics with *L. monocytogenes* without posing the same direct public health risk.

Regional data on *Listeria* contamination and AMR across diverse retail food streams remain limited. This study therefore aimed to (1) quantify baseline *Listeria* prevalence in retail foods, (2) characterize genus diversity and species distribution, and (3) assess phenotypic antimicrobial non-susceptibility. By comparing processed and unprocessed commodities, this work identifies potential environmental reservoirs of resistance and informs regional, risk-based food safety surveillance.

## 2. Materials and Methods

### 2.1. Sample Collection and Study Design

A cross-sectional surveillance study was conducted to determine the prevalence of *Listeria* spp. in retail food products sold in North Carolina, USA. Procurement was designed to capture diverse retail sources, targeting commercial grocery stores, supermarkets, farmers’ markets, and open-air retail outlets across four distinct municipalities in North Carolina, USA: Kannapolis, Concord, Greensboro, and Lexington. These sites represented both urban retail environments and rural agricultural supply chains. All individual food items were purchased at random in their original retail packaging or standard vendor containers to accurately represent consumer exposure points. Following procurement, all samples were immediately placed in insulated coolers equipped with pre-chilled ice packs, transported to the laboratory under continuous cold-chain management (<4 °C), and held under refrigeration to ensure processing commenced within 24 h of collection. Sampling was conducted throughout the study period to account for seasonal variation in food availability. To maintain sample independence, duplicate products, defined as items from the same brand and manufacturer lot number, were excluded during each sampling event. Only one sample of a given product was collected from the same farm outlet on any single operating day.

A total of 861 food samples were analyzed. Commodities targeted for sampling included fresh vegetables, edible fungi (mushrooms), raw meat and dairy products. These specific food categories were selected because they represent well-documented reservoirs or vehicles for *Listeria* contamination. Samples were classified by processing status as unprocessed foods (n = 621), including fresh vegetables, edible mushrooms, raw meat, and raw milk, or processed counterparts (n = 240), including pasteurized milk, cheese, yogurt, and processed meat products. This classification enabled comparison of *Listeria* prevalence between foods not subjected to pathogen-reduction processing and foods that had undergone commercial processing. After purchase, samples were transported to the laboratory in insulated coolers maintained at <4 °C and processed within 2 h of collection.

### 2.2. Isolation and Phenotypic Identification of Listeria spp.

Isolation of *Listeria* species was performed according to standard regulatory enrichment and selective plating methodologies. Depending on the food matrix evaluated, isolation protocols followed the U.S. Food and Drug Administration Bacteriological Analytical Manual (FDA BAM), (for vegetables, edible fungi, and dairy matrices) and the United States Department of Agriculture Food Safety and Inspection Service Microbiology Laboratory Guidebook (USDA-FSIS MLG) (for raw and processed meat matrices).

Samples were homogenized for 1 min using a Seward Stomacher Model 3500 Jumbo Lab Blender (Seward Ltd., West Sussex, UK). Primary enrichment was initiated in Buffered *Listeria* Enrichment Broth (BLEB; Oxoid, Basingstoke, UK) at 30 °C for 4 h without antibiotics. Subsequently, *Listeria* Selective Supplement containing acriflavine (10 mg/L), nalidixic acid (40 mg/L), and cycloheximide (50 mg/L) (Sigma-Aldrich, Steinheim, Germany) was then added, and enrichment continued at 30 °C for 44 h.

After enrichment, a loopful of culture was streaked onto Brilliance™ *Listeria* Selective Agar (Oxoid, Thermo Fisher Scientific, Basingstoke, UK) and Oxford Agar plates (Sigma-Aldrich, Steinheim, Germany), followed by incubation at 35 °C for 48 h. Presumptive positive colonies were selected based on genus-specific phenotypes: those appearing blue-green with opaque halos on the former, and greyish-black to black with black halos on the latter due to esculin hydrolysis.

Colonies with typical morphology were purified by repeated streaking and examined by Gram staining, catalase testing, and molecular confirmation. Catalase testing was performed on overnight cultures using commercial 3% hydrogen peroxide droppers (BBL Catalase Reagent Droppers, BD Diagnostic Systems, Sparks, MD, USA). The immediate evolution of gas bubbles was documented as a positive reaction.

#### Genomic DNA Extraction and Multiplex PCR Identification of *Listeria* Species

Genomic DNA was extracted from purified isolates by boiling lysis. A single colony from an overnight culture was suspended in 100 µL of sterile molecular-grade water and heated at 98–100 °C for 10 min. The lysate was stored at −80 °C for 1 h, thawed at room temperature for 5 min, and centrifuged at 12,700 rpm for 5 min. The supernatant was transferred to sterile microcentrifuge tubes and stored at −20 °C until PCR analysis.

Species identification was performed using a multiplex PCR assay adapted from Mazzette et al. [20]. The assay targeted the genus-specific *prs* gene and species-specific markers for the six classical *Listeria* species: *L. monocytogenes*, *L. ivanovii*, *L. innocua*, *L. seeligeri*, *L. grayi*, and *L. welshimeri*. Primer sequences, target genes, primer concentrations, and expected amplicon sizes were adopted from Mazza et al. and are provided in Table 1.

Assay specificity and run validity were assessed by including positive and negative controls as independent reactions in each multiplex PCR assay. Genomic DNA from reference strains was prepared separately and added to designated positive-control wells to confirm amplification of the expected genus- and species-specific targets under the same reagent composition, primer pool, reaction volume, and thermal cycling conditions used for the test isolates. Positive-control reactions included *L. monocytogenes* ATCC 19115, *L. ivanovii* ATCC 19119, *L. innocua* ATCC 33090, *L. seeligeri* ATCC 35967, *L. grayi* ATCC 19120, and *L. welshimeri* ATCC 35089. A separate no-template control containing sterile molecular-grade water was included in each assay to monitor contamination. Control reactions were not combined with isolate DNA and were interpreted alongside sample reactions before assigning species-level identifications.

PCR amplification was performed using a modified protocol based on Mazza et al. Reactions contained GoTaq^®^ Green Master Mix (Promega Corporation, Madison, WI, USA), 5× GoTaq^®^ Reaction Buffer supplemented with 25 mM MgCl_2_, 10 mM dNTPs, and GoTaq^®^ DNA polymerase (5 U/µL). Primers targeting seven loci (synthesized by Invitrogen Corporation, Carlsbad, CA, USA; Table 2) were resuspended to 10 µM. Template DNA from 73 isolates and 6 control organisms were added at 1.5 µL per reaction. All 14 primers (7 forward and 7 reverse) were combined into a 7-plex primer pool, with each primer adjusted to 2 µM, yielding final primer concentrations of 0.1–2 µM in a 25 µL reaction. A bulk master mix for 92 reactions, including 73 isolates, 6 controls, and 13 additional reactions for pipetting overage and dead volume, was prepared on ice to minimize nonspecific primer-dimer formation. The reaction mixture is summarized in Table 2.

For each reaction, 23.5 µL of bulk master mix was dispensed into a 96-well plate, followed by 1.5 µL of template DNA, control DNA, or nuclease-free water, for a final volume of 25 µL. Thermal cycling conditions were as follows: initial denaturation at 95 °C for 5 min; 35 cycles of denaturation at 94 °C for 45 s, annealing at 60 °C for 45 s, and extension at 72 °C for 60 s; and final extension at 72 °C for 5 min. Amplicons (10 µL) were separated by electrophoresis on a 1.5% (*w*/*v*) agarose gel in 1× TAE buffer containing ethidium bromide at 80 V for 60 min. A 100 bp DNA ladder was loaded in the first and last lanes of each tier to estimate fragment sizes between 201 and 749 bp. Bands were visualized under UV illumination. Isolates positive for *prs* but negative for all species-specific targets were classified as unidentified *Listeria* spp.

### 2.3. Antibiotic Susceptibility Testing

Antimicrobial susceptibility was determined using the Kirby–Bauer disk diffusion method according to Clinical and Laboratory Standards Institute (CLSI) guidelines. Fifteen antimicrobial agents representing nine classes were tested (Table 3). Because CLSI interpretive criteria are not available for *Listeria* spp., inhibition-zone breakpoints for *Staphylococcus* spp. and *Enterococcus* spp. were applied as surrogate criteria, as summarized in Table 3. *Staphylococcus* aureus ATCC 13301 and methicillin-resistant *S. aureus* ATCC 700698, recommended in CLSI M100 [21], were used as quality-control organisms. After incubation at 35 °C for 18–24 h, inhibition-zone diameters were measured and interpreted as susceptible, intermediate, or resistant according to CLSI M100 Performance Standards for Antimicrobial Susceptibility Testing [21]. Multidrug resistance was defined as non-susceptibility to at least one antimicrobial agent in three or more antimicrobial categories [22].

### 2.4. Statistical Analysis

Statistical analyses were performed using GraphPad Prism version 11.0.12 (GraphPad Software, San Diego, CA, USA). Prevalence was calculated for each food subtype and macro-category as the percentage of positive samples among the total number tested in that group. Overall and pairwise differences in bacterial prevalence among food categories and between processed and unprocessed food matrices were evaluated using two-tailed Fisher’s exact tests. Associations were estimated using relative risks, odds ratios, and corresponding 95% confidence intervals. The Relative Risk (RR) was calculated as:

$$RR=\frac{Unprocessed\;Prevalence\;(\%)}{Processed\;Prevalence\;(\%)}$$

The Odds Ratio (OR) was calculated as:

$$OR=\frac{Unprocessed\;Positives\;x\;Processed\;Negatives}{Unprocessed\;Negatives\;x\;Processed\;Positives}$$

For antimicrobial susceptibility analyses, resistant and intermediate isolates were grouped as non-susceptible, and isolates non-susceptible to at least one agent in three or more therapeutic classes were classified as multidrug resistant. Statistical significance was set at *p* < 0.053.

## 3. Results

### 3.1. Prevalence and Distribution Listeria Species

**Figure 1:**
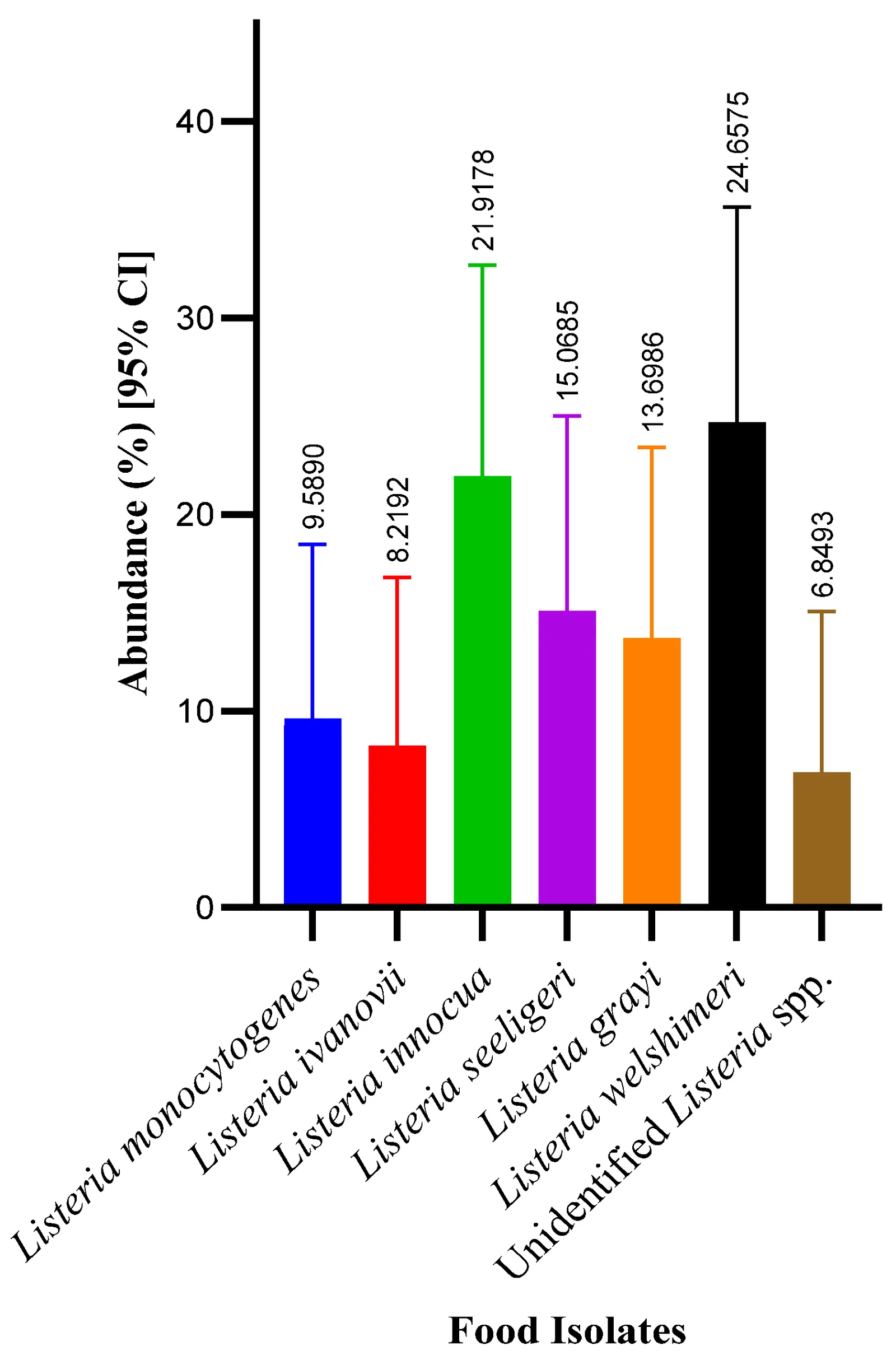
Species Diversity and Abundance of Isolates (n = 73). Error Bars—95% Confidence Interval.

Out of 861 retail and farm-gate food samples analyzed (including vegetables, edible fungi (mushrooms), raw and processed meats, raw and processed dairy), 73 (8.48%) were positive for *Listeria* spp. Multiplex PCR 68 of these isolates (93.15% to the species level leaving five (6.85% unidentified (Figure 1).

Among the identified taxa, *Listeria welshimeri* was recovered most frequently, followed by *L. innocua*, *L. seeligeri* and *L. grayi* (Figure 1). The pathogenic species were notably less common within the total isolate collection with *L. monocytogenes* and *L. ivanovii* 8–9% of the recovered genus population. Crucially, all pathogenic isolates were restricted to raw food commodities.

Non-pathogenic environmental species predominated, exhibiting an overall of 7.08% prevalence across sampled foods (Table A2). Conversely, pathogenic species (*L. monocytogenes* and *L. ivanovii*) were less frequent, accounting for 17.81% of total isolates and appearing in 1.51% of all evaluated food samples (Table A2).

### 3.2. Product-Level Distribution of Listeria spp.

Analysis of individual food commodities revealed considerable variation in contamination rates (Table 4). Among fresh vegetable produce, the highest prevalence was observed in kale (22.22%, 8/36), followed by Alfalfa sprouts (17.39%, 4/23), romaine lettuce (16.67%, 6/36) and spinach (13.33%, 8/60). Within the raw meat products, raw pork exhibited the highest prevalence (20.00%, 2/10), followed by ground beef (13.89%, 5/36) and raw chicken (9.52%, 4/42) (Table 4).

In contrast, recovery of *Listeria* spp. from processed foods was rare. No *Listeria* isolates were detected in processed meat products including pork ham, pork sausage, beef hotdogs, and smoked pork (0/39). Similarly, pasteurized milk products and ten of the eleven cheese varieties assessed were free of contamination. The only positive processed food sample was cream cheese, which exhibited a prevalence of 7.14% (1/14) (Table 4 and Table A1).

#### 3.2.1. Effect of Food Processing on *Listeria* Contamination

**Figure 2:**
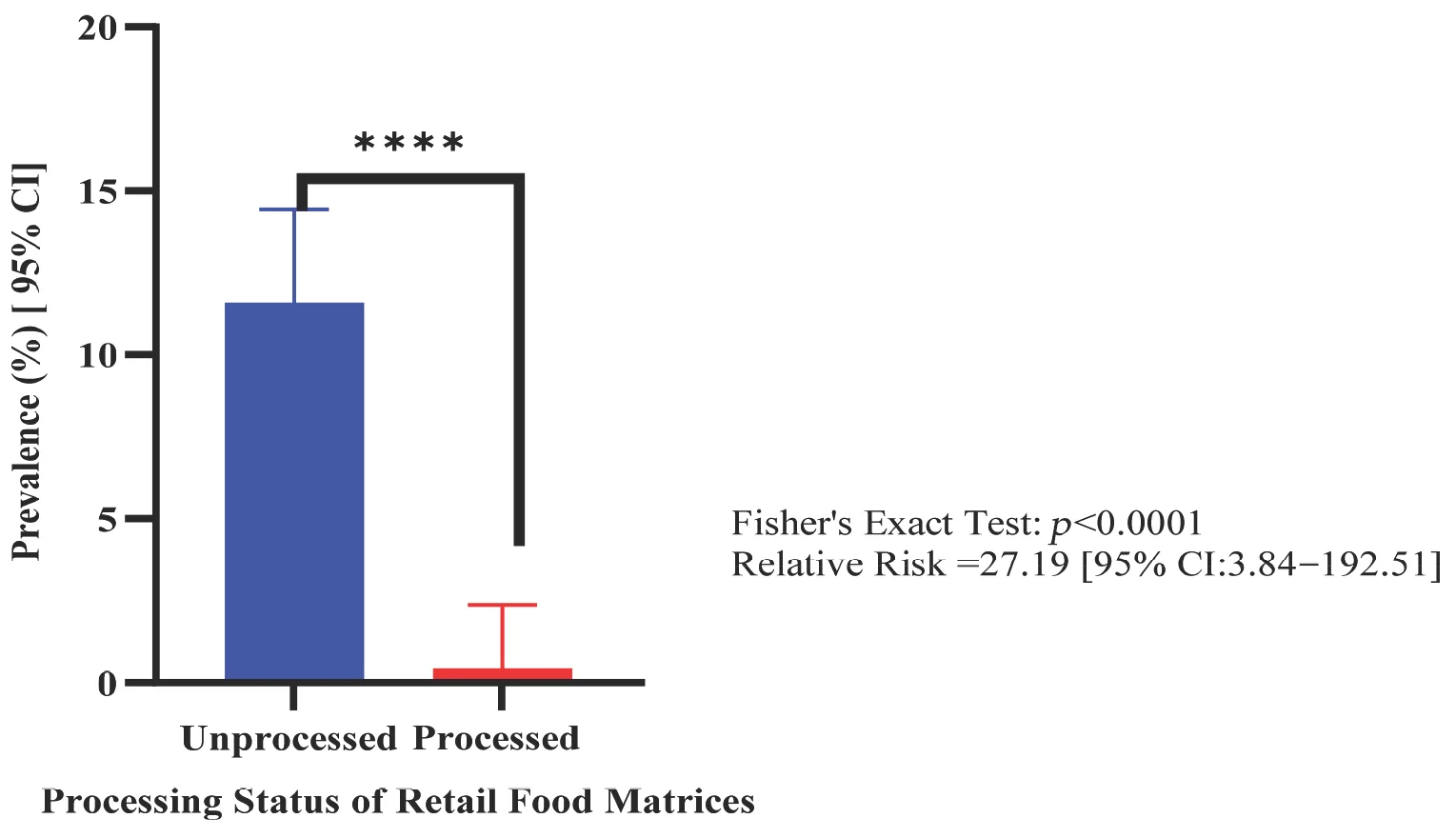
Comparison of *Listeria* spp. Prevalence and Relative Risk Metrics Retail Food Categories (N = 861). Error bars = 95% Confidence Interval (CI). Statistical analysis was performed using Fisher’s exact test; (****) indicate highly significant difference *p* < 0.00001.

Unprocessed foods exhibited a significantly higher prevalence of *Listeria* spp., compared to processed alternatives (Fisher’s exact test, *p* < 0.0001; Figure 2). Risk metrics confirmed a strong positive association between raw processing status and contamination risk. Specifically, unprocessed food matrices were approximately 27 times more likely to harbor the pathogen, demonstrating a pronounced vulnerability to contamination when compared directly to processed items.

#### 3.2.2. Prevalence of *Listeria* spp. Across Major Food Categories

**Figure 3:**
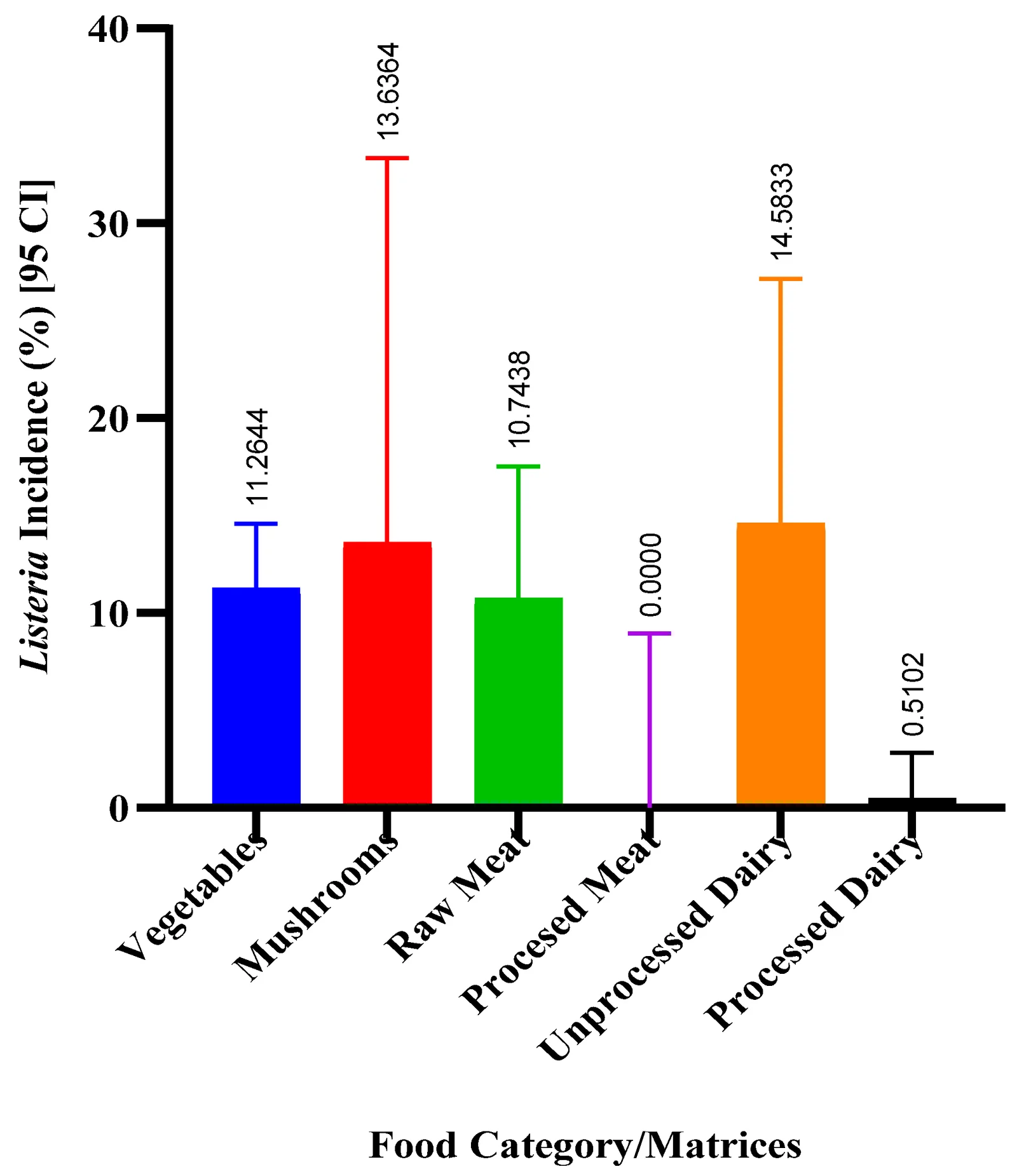
Distribution and Baseline Prevalence of *Listeria* spp. Across Key Retail Food Categories. Error bars represent 95% confidence intervals. Statistical significance was determined at *p* < 0.05.

*Listeria* spp. contamination varied across the evaluated matrices, peaking in raw milk followed by mushrooms, vegetables, and raw meats (Figure 3). Conversely, processed dairy products exhibited a minimal contamination rate, while processed meat products remained entirely free of the pathogen. Pairwise comparisons revealed that the higher prevalence observed in vegetables was statistically significant when contrasted directly against processed dairy products (*p* = 0.0017; Figure 3).

When categorized by pathogenic potential, non-pathogenic *Listeria* species predominated over pathogenic species across the evaluated food matrices (Table A3). Vegetables harbored both groups, though non-pathogenic strains were recovered at a substantially higher frequency than pathogenic ones. Similarly, raw meat was dominated by non-pathogenic species, which occurred at nearly three times the rate of their pathogenic counterparts. In contrast, raw milk exhibited a more balanced distribution, with pathogenic and non-pathogenic species isolated at comparable frequencies. Notably, mushrooms yielded non-pathogenic *Listeria* exclusively, with no pathogenic species recovered from this matrix (Table A3).

### 3.3. Overall Antimicrobial Resistance Profile

The evaluated *Listeria* isolates exhibited variable susceptibility patterns across the tested panel of therapeutic agents (Table 5). Phenotypic resistance was highest for Penicillin G, followed by clindamycin, levofloxacin, and ciprofloxacin. Intermediate susceptibility was most frequently observed for levofloxacin, followed by chloramphenicol. Conversely, tobramycin, tetracycline, and trimethoprim–sulfamethoxazole demonstrated exceptional efficacy, with almost all recovered isolates remaining fully susceptible to these antimicrobials (Table 5).

#### 3.3.1. Species-Specific Antibiotic Resistance Patterns

Species-specific antimicrobial resistance profiles varied considerably across the genus collection (Table A4). All *L. monocytogenes* isolates remained fully susceptible to amikacin, azithromycin, tetracycline, tobramycin, and trimethoprim–sulfamethoxazole (Table A4). However, phenotypic resistance to penicillin G and levofloxacin was noted within this pathogenic group. Similarly, *L. ivanovii* exhibited extensive resistance to nitrofurantoin, penicillin G, and levofloxacin (Table A4).

Among the non-pathogenic species, distinct resistance profiles were observed across different taxa (Table A4). *L. innocua* displayed resistance to kanamycin and penicillin G, while *L. welshimeri* frequently exhibited intermediate susceptibility to levofloxacin, moxifloxacin, and azithromycin. Notably, *L. grayi* demonstrated the highest baseline resistance levels among the non-pathogenic isolates, particularly toward penicillin G, clindamycin, and levofloxacin.

#### 3.3.2. Non-Susceptibility Patterns Across Pathogenic and Non-Pathogenic *Listeria* spp.

When resistant and intermediate strains were pooled into a unified non-susceptible cohort, most tested antimicrobials exhibited comparable profiles between pathogenic and non-pathogenic *Listeria* isolates (Table 6). Observed variations in non-susceptibility rates for penicillin G, nitrofurantoin, and levofloxacin between the two cohorts were not significantly different (*p* > 0.05). Conversely, clindamycin non-susceptibility was significantly higher among pathogenic isolates (*p* = 0.0175; Table 6). In contrast, azithromycin non-susceptibility was restricted exclusively to non-pathogenic isolates (*p* = 0.0461).

#### 3.3.3. Multidrug Resistance Profiles of *Listeria* Phenotypes

Following established criteria, multidrug resistance (MDR) was defined as non-susceptibility to at least one antimicrobial agent in three or more therapeutic classes (Table 7). More than half of the total isolate collection was classified as MDR, with a higher prevalence observed among pathogenic strains than non-pathogenic counterparts. At the species level, MDR was identified in a clear majority of *L. monocytogenes* isolates and was universal among all *L. ivanovii* strains (Table 7).

Among the non-pathogenic environmental species, MDR distribution varied significantly by taxon. The phenotype was universal in *L. grayi*, followed in descending frequency by *L. innocua*, *L. seeligeri*, and *L. welshimeri* (Table 7). Collectively, these findings indicate that while multidrug resistance is widely distributed across the genus, the overall burden remains highest within pathogenic lineages.

## 4. Discussion

*Listeria* species remain important foodborne microorganisms because of their ability to persist in diverse environmental niches and contaminate foods throughout the production chain. Among them, *L. monocytogenes* is the principal human pathogen and a major concern in ready-to-eat (RTE) foods, which are particularly high-risk due to absence of terminal listericidal treatment step and the organisms’ capacity to survive and proliferate under conditions that inhibit many other pathogens [11]. This study investigated the occurrence, species diversity, distribution, and antimicrobial resistance profiles of *Listeria* spp. recovered from retail and farm-gate foods in North Carolina, USA. Overall, contamination was widespread in raw commodities, particularly vegetables, raw dairy products, and meats, while processed foods showed markedly lower prevalence, highlighting the effectiveness of post-harvest interventions. Although non-pathogenic species predominated, the detection of *L. monocytogenes* and *L. ivanovii* in raw food matrices underscores their continued relevance as potential sources of human exposure. Furthermore, the high prevalence of antimicrobial resistance (AMR) and multidrug resistance (MDR) across both pathogenic and environmental *Listeria* isolates suggests that food production environments may act as important reservoirs of resistance determinants. Collectively, these findings support the need for integrated food safety and AMR surveillance within a One Health framework.

### 4.1. Diversity and Distribution of Listeria Species in Retail Foods

The adapted multiplex PCR assay identified 68 of 73 presumptive *Listeria* isolates (93.2%), indicating its suitability for routine species-level identification of classical *Listeria* species. The five unidentified isolates may represent species outside the multiplex primer panel or isolates with sequence variation that reduced primer binding efficiency, a recognized limitation of targeted molecular assays [23,24]. Similar limitations have been reported previously, supporting the complementary use of higher-resolution approaches such as multi-locus sequence typing (MLST) and whole-genome sequencing (WGS) for comprehensive taxonomic characterization [16]. Differences in identification efficiency may be attributable to variations in sample type, species diversity, primer specificity, and the presence of atypical strains.

The overall prevalence of *Listeria* spp. in retail and farm-gate foods was 8.48% (73/861), with *L. monocytogenes* accounting for less than 1% of all samples. Although lower than that reported in several multi-state U.S. surveys, these differences are likely attributable to geographical location, commodity type, sampling design, and environmental conditions [25]. Consistent with previous studies, non-pathogenic *Listeria* species predominated, reflecting their adaptation to agricultural and food-processing environments [25,26]. Interestingly, *L. welshimeri* (2.08% prevalence) was the most frequently recovered species, whereas many previous investigations have identified *L. innocua* as the dominant environmental species but currently at 1.85% [27]. This variation may reflect regional differences in environmental reservoirs, agricultural practices, or food matrices. Nevertheless, the recovery of *L. monocytogenes* and *L. ivanovii* from foods intended for human consumption confirms the continued circulation of pathogenic *Listeria* within the food supply and reinforces the importance of effective surveillance and hygienic practices throughout the farm-to-fork continuum [28]. The high relative frequency of both *L. welshimeri* and *L. innocua* in North Carolina retail food channels provides food safety managers with a broader, more statistically robust data pool for tracking sanitation failures and mapping facility harborages before *L. monocytogenes* colonization can occur. Although *L. innocua* was not the predominant species in this study, its frequent recovery supports its continued value as an environmental indicator organism. Because *L. innocua* occupies ecological niches like those of *L. monocytogenes*, its detection can provide an early indication of sanitation deficiencies before pathogenic *Listeria* becomes established. Consequently, environmental monitoring programs that include non-pathogenic *Listeria* species remain an important component of preventive food safety systems [27].

### 4.2. Impact of Food Processing on Listeria Contamination

Food processing had a pronounced effect on *Listeria* occurrence. Raw commodities exhibited substantially higher contamination than processed foods, with processing reducing the likelihood of contamination by approximately 27-fold. These findings reinforce raw commodities as primary reservoirs of contamination. In contrast, processed foods showed minimal contamination, with only one positive processed dairy sample and no detections in processed meat, confirming the effectiveness of thermal processing, pasteurization, and HACCP-based interventions [28,29,30].

Fresh vegetables contributed to the largest number of positive samples, with leafy vegetables such as kale and collards showing particularly high contamination frequencies [31,32,33]. Similar observations have been reported globally, where contamination of fresh produce has been associated with irrigation water, soil, manure, wildlife, and post-harvest handling practices [14,34,35,36]. Raw milk exhibited very high prevalence among the commodity groups examined, consistent with studies describing contamination through infected animals, contaminated milking equipment, or environmental exposure during milk production [37]. Because these products are often consumed raw, they present a direct route for exposure. Raw meat contamination (10.74%) was consistent with global retail surveys [38], while relatively higher prevalence observed in pork may reflect contamination during slaughter and carcass processing [39]. Although *L. monocytogenes* has previously been isolated from mushroom production environments, its absence in mushroom samples in the present study may reflect differences in cultivation practices or local environmental conditions [40,41].

The absence of *Listeria* from processed meat products and the very low prevalence observed in processed dairy products demonstrate the effectiveness of validated processing interventions. The single positive processed dairy sample likely represents post-processing contamination rather than process failure. Collectively, these findings support strengthening preventive controls within the raw supply chain while maintaining effective environmental monitoring programs to minimize opportunities for post-processing contamination.

### 4.3. Distribution of Pathogenic and Non-Pathogenic Species

Non-pathogenic *Listeria* species predominated across all food categories, whereas pathogenic species were recovered exclusively from raw commodities. This distribution agrees with previous reports describing environmental *Listeria* species as common inhabitants of agricultural ecosystems while pathogenic species occur less frequently but pose substantially greater public health risks [6,42,43].

The detection of *L. monocytogenes* in fresh vegetables, and raw milk is particularly important because these commodities may be consumed with little or no additional pathogen reduction. The ability of *L. monocytogenes* to survive refrigeration, tolerate environmental stresses, and persist within food-processing environments further increases its significance as a foodborne pathogen [11,14,36]. Likewise, the occurrence of *L. ivanovii*, although less frequently associated with human disease, demonstrates that multiple pathogenic *Listeria* species remain present within the food supply [14,36]. Although *L. ivanovii* is traditionally classified as a veterinary pathogen causing ruminant abortions, it is increasingly recognized as an opportunistic human pathogen capable of causing severe systemic listeriosis in immunocompromised individuals. Its presence in fresh retail matrices indicates a breakdown in pre-harvest biosecurity, likely driven by animal manure contamination or agricultural runoff bypassing standard Good Agricultural Practices (GAPs). Overall, these findings support risk-based surveillance strategies incorporating commodity-specific hazard analyses together with routine environmental monitoring to identify contamination sources before products reach consumers.

### 4.4. Antimicrobial Resistance Profiles

The emergence of antimicrobial resistance (AMR) in *Listeria* species recovered from retail foods threatens standard clinical treatments for human listeriosis. Historically, the genus *Listeria* has been considered uniformly susceptible to β-lactams and aminoglycosides. Our study on phenotypic antimicrobial susceptibility testing demonstrated resistance to multiple clinically important antimicrobial classes among foodborne *Listeria* isolates. There was broad reduced susceptibility which was most frequently observed to Penicillin G, levofloxacin, clindamycin, and ciprofloxacin, whereas tetracycline, tobramycin, and trimethoprim–sulfamethoxazole remained highly effective against most isolates. Differences between the resistance profiles observed in this study and those reported elsewhere likely reflect regional variation in antimicrobial usage, agricultural practices, and the composition of circulating *Listeria* populations [17,44]. The presence of overlapping resistance profiles in non-pathogenic *L. innocua* and *L. welshimeri* against clinically vital classes (e.g., fluoroquinolones, macrolides, and aminoglycosides) indicates a shared resistome within food processing microenvironments.

Reduced susceptibility to Penicillin G is of particular concern because β-lactam antibiotics remain the cornerstone of treatment for invasive listeriosis. Although clinically significant resistance remains relatively uncommon, increasing reports of reduced susceptibility among food and environmental isolates suggest that agricultural environments may represent important reservoirs of resistant *Listeria* [19,44,45,46]. Likewise, reduced susceptibility to fluoroquinolones may indicate antimicrobial selection pressures operating throughout food production systems [47,48].

Resistance profiles differed considerably among species. *Listeria ivanovii* exhibited the greatest resistance among the pathogenic species, whereas *L. grayi* displayed the highest resistance burden among the non-pathogenic species. These observations indicate substantial interspecies variability and support the hypothesis that environmental *Listeria* populations contribute to the persistence and dissemination of antimicrobial resistance within food-production environments [49,50].

Multidrug resistance was common, occurring in 64.71% of all isolates and more frequently among pathogenic species than non-pathogenic species. Although the present study relied solely on phenotypic susceptibility testing, the widespread occurrence of multidrug resistance suggests that food production environments may serve as reservoirs of resistant *Listeria* [51,52]. Because non-pathogenic *Listeria* species occupy ecological niches like those of *L. monocytogenes*, monitoring their antimicrobial resistance profiles may provide valuable insight into resistance selection within food-production environments. Future investigations incorporating whole-genome sequencing will be essential to determine whether these phenotypes result from intrinsic resistance mechanisms, acquisition of resistance genes, or horizontal gene transfer within food-associated microbial communities.

### 4.5. Study Limitations

Several limitations should be considered when interpreting these findings. First, the surveillance was limited to selected regions of North Carolina and therefore may not represent broader U. S. food supply. Second, the cross-sectional design provides only a temporal snapshot of contamination during the study period. Third, the relatively small number of pathogenic isolates limited the statistical power or species-specific comparisons. Fourth antimicrobial susceptibility was evaluated using phenotypic methods without molecular characterization of resistance genes, serotypes or genomic lineages. Consequently, genetic basis of the observed resistance profiles could not be determined. Finally, several food commodities associated with listeriosis outbreaks, including fruits and Mexican-style soft cheeses, e.g., queso fresco, were not included and will be incorporated into future surveillance studies.

High MDR levels among environmental species further suggest that food production environments may facilitate maintenance and dissemination of resistance determinants [51,52]. The elevated rates of phenotypic Penicillin G resistance observed among the non-pathogenic species, such as *L. innocua*, suggest that these food-associated strains may act as phenotypic reservoirs of resistance. While existing literature heavily establishes that non-pathogenic *Listeria* can share resistance determinants via horizontal gene transfer (HGT) within food matrices, no molecular or genomic characterization was performed in the present study to confirm the specific underlying resistance genes or active transfer mechanisms. Therefore, these phenotypic trends warrant future genotypic investigations to confirm the precise genetic elements responsible for this β-lactam resistance.

## 5. Conclusions

This study provides baseline data on the prevalence, species diversity, and antimicrobial resistance of *Listeria* spp. in retail and farm-gate foods in some regions in North Carolina, USA. Overall prevalence was 8.55%, with contamination concentrated on raw commodities, particularly milk, vegetables, mushrooms, and meats. Processed foods showed minimal contamination, confirming the effectiveness of post-harvest interventions. Molecular identification revealed a predominance of non-pathogenic species, while pathogenic *L. monocytogenes* and *L. ivanovii* were confined to raw foods, underscoring ongoing exposure risks. In addition, widespread antimicrobial resistance and high MDR prevalence across both pathogenic and environmental isolates indicate that food systems may serve as important reservoirs of resistance determinants. These findings highlight the need for continued surveillance of *Listeria* spp. beyond *L. monocytogenes*, strengthened farm-to-fork control strategies, and integrated monitoring of antimicrobial resistance within a One Health framework to mitigate foodborne and resistance-related risks.

## Figures and Tables

**Table 1:** Oligonucleotide primer sequences and diagnostic amplicon configurations utilized for multiplex PCR speciation.

Species/ Target Gene	Primer Name	Primer Sequence (5′ → 3′)	Product Size (bp)	Final Primer Concentration (µM)
*Listeria* Genus (*prs*)	prs-F prs-R	GCTGAAGAGATTGCGAAAGAAG CAAAGAAACCTTGGATTTGCGG	370	0.40
*L. monocytogenes* (*Lmo1030*)	Lmo1030F Lmo1030R	GCTTGTATTCACTTGGATT GTCTGG ACCATCCGCATATCTCAGCCAACT	509	0.56
*L. ivanovii* (namA)	liv22228F liv22228R	CGAATTCCTTATTCACTTGAGC GGTGCTGCGAACTTAACTCA	463	0.52
*L. innocua* (Lin0464)	Lin0464F Lin0464R	CGCATTTATCGCCAAAACTC TGCTGACATAGACGCGATTG	749	2.5
*L. seeligeri* (*Lmo0333*)	lseelinF lseelinR	GTACCTGCTGGAGTACATA CTGTCTCCATATCCGTACAG	673	1.36
*L. grayi* (*Oxidoreductase*)	JOgrayiF JOgrayiR	GCGGATAAAGGTGTTCGGTCAA ATTTGCTATCGTCCGAGGCTAGG	201	0.24
*L. welshimeri* (*scrA*)	Lwe1801F Lwe1801R	CGTGGCACAATAGCAATCTG GACATGCCTGCTGAACTAGA	281	2.5

**Table 2:** The components of PCR master mix used for identification of presumptive *Listeria* spp.

Component	Volume for Single Run (µL)	Final Concentration
5× GoTaq^®^ Master Mix	5	1×
MgCl2 Solution (25 mM)	1.5	3.0 mM total
dNTP Mix (10 mM each)	0.5	400 µM each
GoTaq^®^ DNA Polymerase (5 U/µL)	0.125	0.625 U extra
7 Primer Sets (pooled)	~3.5	0.1 µM to 0.2 µM each
Nuclease-Free Water	12.875	N/A
Subtotal Aliquoted per well	23.5	1 U
DNA template	1.5	-
Total	25.0	<100 ng per well

N/A = Not Applicable.

**Table 3:** Zone of Inhibition Interpretive Criteria for *Listeria* spp., Based on Surrogate Breakpoints.

Drug Class	Antibiotic	Disk Potency (µg)	Surrogates	S (mm)	I (mm)	R (mm)
Penicillins	Penicillin G	10 U	*Enterococcus* spp.	≥15	-	≤14
Aminoglycosides	Amikacin	30	*Staphylococcus* spp.	≥17	15–16	≤14
	Kanamycin	30		≥18	14–17	≤13
	Tobramycin	10		≥15	13–14	≤12
Macrolides	Erythromycin	15	*Staphylococcus* spp.	≥23	14–22	≤13
	Azithromycin	15		≥18	14–17	≤13
Fluoroquinolones	Ciprofloxacin	5	*Staphylococcus* spp.	≥21	16–20	≤15
	Levofloxacin	5		≥19	16–18	≤15
	Moxifloxacin	5		≥24	21–23	≤20
Phenicols	Chloramphenicol	30	*Staphylococcus* spp.	≥18	13–17	≤12
Lincosamides	Clindamycin	2	*Staphylococcus* spp.	≥21	15–20	≤14
Rifamycins	Rifampin	5	*Staphylococcus* spp.	≥20	17–19	≤16
Tetracyclines	Tetracycline	30	*Staphylococcus* spp.	≥19	15–18	≤14
Folate Pathway	Trimethoprim	5	*Staphylococcus* spp.	≥16	11–15	≤10
	Trimethoprim–Sulfamethoxazole	1.25/23.75		≥16	11–15	≤10

**Table 4:** Prevalence and Species Distribution of *Listeria* spp. in Selected Food Categories.

Food Category and Individual Item Subtype	Total Samples	*L. monocytogenes* (n = 7)	*L. ivanovii* (n = 6)	*L. innocua* (n = 16)	*L. seeligeri* (n = 11)	*L. grayi* (n = 10)	*L. welshimeri* (n = 18)	Unknown *Listeria* (n = 5)	Overall Prevalence N = 73
VEGETABLES	435	0.46	1.15	2.76	1.61	1.84	2.99	0.46	11.26 (49)
Cucumber	58	—	1.72	—	—	—	1.72	3.45	6.90
Lettuce	22	—	—	4.55	—	—	—	—	4.55
Alfalfa Sprouts	23	4.35	—	4.35	—	4.35	4.35	—	17.39
Kale	36	—	2.78	5.56	2.78	8.34	2.78	—	22.22
Green onion	45	—	2.22	2.22	2.22	—	4.45	—	11.11
Spinach	60	—	—	3.33	5.00	1.66	3.33	—	13.33
Collard green	19	5.26	5.26	—	—	—	5.26	—	15.79
Romaine lettuce	36	—	—	2.78	2.78	5.56	5.56	—	16.67
Cilantro	24	—	—	8.33	—	—	4.17	—	12.50
Mixed lettuce	9	—	—	—	—	—	11.11	—	11.11
Carrot	9	—	—	—	—	—	—	—	—
Mixed vegetables	40	—	2.50	—	—	—	2.50	—	5.00
Tomato	54	—	—	3.70	1.85	1.85	—	—	7.41
EDIBLE FUNGI (Mushrooms)	22	—	—	9.09	4.55	—	—	—	13.64
RAW MEAT	121	2.48	—	1.65	1.65	1.65	2.48	0.83	10.74
Raw Ground Beef	36	2.78	—	—	2.78	—	5.56	2.78	13.89
Raw Beef	30	—	—	3.33	—	—	3.33	—	6.67
Raw Chicken	42	2.38	—	2.38	2.38	2.38	—	—	9.52
Ground Pork	3	—	—	—	—	—	—	—	—
Raw Pork	10	10.00	—	—	—	10.00	—	—	20.00
UNPROCESSED DAIRY (Milk)	48	4.17	2.08	—	2.08	—	4.17 (2)	2.08	14.58
PROCESSED DAIRY	196	—	—	—	—	—	—	0.51	0.51
Cream Cheese	14	—	—	—	—	—	—	7.14	7.14
Other Processed Dairy ^a^	*182*	—	—	—	—	—	—	—	—
TOTAL STUDIED POOL	861	0.81	0.69	1.85	1.27	1.15	2.08	0.58	8.48

Note: N = total samples; n = positive samples; dashes (—) indicate zero detection (0.00%). ^a^ Omitted due to 0% prevalence: Processed Meats (Pork Ham, Pork Sausage, Beef Hotdog, Smoked Pork; N = 39) and 11 Processed Dairy items (Whole Milk, 2% Milk, Mozzarella, Sandwich, Pepper Jack, Snack, Parmesan, Skimmed Milk, Cheddar, Feta, Yogurt; N = 182). Full individual distributions are catalogued in Appendix A—Table A1.

**Table 5:** Overall Phenotypic Distribution and Antibiotic Resistance Profiles of Recovered *Listeria* Isolates (n = 68).

Antibiotics	Resistant (R) % (n)	Intermediate (I) % (n)	Susceptible (S) % (n)
Amikacin	0.00 (0)	4.41 (3)	95.59 (65)
Azithromycin	7.35 (5)	13.24 (9)	79.41 (54)
Chloramphenicol	10.29 (7)	16.18 (11)	73.53 (50)
Ciprofloxacin	14.71 (10)	14.71 (10)	70.59 (48)
Clindamycin	23.53 (16)	13.24 (9)	63.24 (43)
Erythromycin	7.35 (5)	7.35 (5)	85.29 (58)
Kanamycin	26.47 (18)	23.53 (16)	50.00 (34)
Levofloxacin	22.06 (15)	32.35 (22)	45.59 (31)
Moxifloxacin	17.65 (12)	11.76 (8)	70.59 (48)
Penicillin G	39.71 (27)	0.00 (0)	60.29 (41)
Rifampin	14.71 (10)	5.88 (4)	79.41 (54)
Tetracycline	1.47 (1)	0.00 (0)	98.53 (67)
Tobramycin	1.47 (1)	0.00 (0)	98.53 (67)
Trimethoprim	19.12 (13)	5.88 (4)	75.00 (51)
TMP-SMX ^a^	1.47 (1)	0.00 (0)	98.53 (67)

^a^ TMP-SMX, trimethoprim–sulfamethoxazole.

**Table 6:** Comparative non-susceptibility/resistance of pathogenic and non-pathogenic *Listeria* isolates.

Antibiotic Screening Panel	Pathogenic Cohort (N = 3) ^a^ Non-Susceptible Frequency % (n)	Non-Pathogenic Cohort (N = 55) ^b^ Non-Susceptible Frequency % (n)	Two-Sided Fisher’s Exact *p*-Value	Significance Summary
Amikacin	7.69 (1) [1.37–33.31]	3.64 (2) [1.00–12.32]	0.4497	ns
Azithromycin	0.00 (0) [0.00–22.81]	25.45 (14) [15.81–38.30]	0.0461	*
Chloramphenicol	30.77 (4) [12.63–58.29]	14.55 (8) [7.56–26.16]	0.1332	ns
Ciprofloxacin	23.08 (3) [8.18–50.25]	32.73 (18) [21.81–45.90]	0.7423	ns
Clindamycin	58.85 (7) [29.13–76.79]	23.64 (13) [11.55–32.37]	0.0175	*
Erythromycin	7.69 (1) [1.37–33.31]	16.36 (9) [8.86–28.26]	0.6726	ns
Kanamycin	30.77 (4) [12.63–58.29]	50.91 (28) [38.08–63.62]	0.2227	ns
Levofloxacin	46.15 (6) [23.21–70.87]	43.64 (24) [31.37–56.73]	0.9999	ns
Moxifloxacin	15.38 (2) [4.33–42.23]	30.91 (17) [20.28–44.03]	0.3341	ns
Penicillin G	61.54 (8) [35.53–82.28]	34.55 (19) [23.36–47.75]	0.0768	ns
Rifampin	23.08 (3) [8.18–50.25]	16.36 (9) [8.86–28.26]	0.6702	ns
Tetracycline	0.00 (0) [0.00–22.81]	1.82 (1) [0.32–9.61]	>0.9999	ns
Tobramycin	0.00 (0) [0.00–22.81]	1.82 (1) [0.32–9.61]	>0.9999	ns
Trimethoprim	15.38 (2) [4.33–42.23]	23.64 (13) [14.47–36.35]	0.7180	ns
TMP-SMX ^c^	0.00 (0) [0.00–22.81]	1.82 (1) [0.32–9.61]	>0.9999	ns

^a^ Pathogenic isolates included *Listeria monocytogenes* (n = 7) and *Listeria ivanovii* (n = 6). ^b^ non-pathogenic isolates included *Listeria innocua* (n = 16), *Listeria seeligeri* (n = 11), *Listeria grayi* (n = 10), and *Listeria welshimeri* (n = 18). * *p* < 0.05; ns, not significant. ^c^ TMP-SMX, trimethoprim–sulfamethoxazole.

**Table 7:** Distribution of multidrug-resistant phenotypes among classical *Listeria* isolates (n = 68).

Taxonomy and Species Line	Total Isolates Tested (*N*)	Multidrug Resistant Isolates ^a^ Count (*n*)	Species-Specific MDR Incidence (%)	Primary Phenotypic Co-Resistance Signature Tracks
Pathogenic Cohort	13	11	84.62 [57.77–97.23]	-
*L. monocytogenes*	7	5	71.43 [35.89–94.92]	β-Lactams, Fluoroquinolones, Lincosamides
*L. ivanovii*	6	6	100.00 [60.57–100.00]	β-Lactams, Fluoroquinolones, Aminoglycosides
Non-Pathogenic Cohort	55	33	60.00 [46.81–71.88]	-
*L. welshimeri*	18	4	22.22 [8.50–45.06]	β-Lactams, Fluoroquinolones, Aminoglycosides
*L. innocua*	16	12	75.00 [50.50–89.83]	β-Lactams, Fluoroquinolones, Aminoglycosides
*L. seeligeri*	11	7	63.64 [35.84–84.83]	β-Lactams, Fluoroquinolones, Aminoglycosides
*L. grayi*	10	10	100.00 [72.25–100.00]	β-Lactams, Fluoroquinolones, Lincosamides
Combined Genus Total	68	44	64.71 [52.84–75.00]	Overarching Retail Resistome Saturation Footprint

^a^ Multidrug resistance (MDR) is defined as phenotypic resistance to at least one antimicrobial agent across three or more distinct structural classes.

## Data Availability

The original contributions presented in this study are included in the article. Further enquiries can be directed at the corresponding author.

## Abbreviations

The following abbreviations are used in this manuscript:

AMR	Antimicrobial Resistance
MDR	Multidrug Resistance
TMP-SMX	Trimethoprim–sulfamethoxazole.
BLEB	Buffered *Listeria* Enrichment Broth
TAE Buffer	Tris–acetate–EDTA Buffer

## Appendix A

**Table A1:** Distribution and Prevalence of *Listeria* species in fresh produce, meat and dairy samples from farms and retail outlets (n = 866).

Food Category and Individual Item Subtype	Total Number of Samples (*N*)	*L. mono* * % (n)	*L. ivanovii* % (n)	*L. innocua* % (n)	*L. seeligeri* % (n)	*L. grayi* % (n)	*L. welshimeri* % (n)	Unknown *Listeria* spp. % (n)	Overall Prevalence % (n)
VEGETABLES	435	0.46 (2)	1.15 (5)	2.76 (12)	1.61 (7)	1.84 (8)	2.99 (13)	0.46 (2)	11.49 (49)
Cucumber	58	0.00 (0)	1.72 (1)	0.00 (0)	0.00 (0)	0.00 (0)	1.72 (1)	3.45 (2)	6.90 (4)
Lettuce	22	0.00 (0)	0.00 (0)	4.55 (1)	0.00 (0)	0.00 (0)	0.00 (0)	0.00 (0)	4.55 (1)
Alpha Sprouts + Alfalfa	23	4.35 (1)	0.00 (0)	4.35 (1)	0.00 (0)	4.35 (1)	4.35 (1)	0.00 (0)	17.39 (4)
Kale	36	0.00 (0)	2.78 (1)	5.56 (2)	2.78 (1)	8.34 (3)	2.78 (1)	0.00 (0)	22.22 (8)
Green onion	45	0.00 (0)	2.22 (1)	2.22 (1)	2.22 (1)	0.00 (0)	4.45 (2)	0.00 (0)	11.11 (5)
Spinach	60	0.00 (0)	0.00 (0)	3.33 (2)	5.00 (3)	1.66 (1)	3.33 (2)	0.00 (0)	13.33 (8)
Collard green	19	5.26 (1)	5.26 (1)	0.00 (0)	0.00 (0)	0.00 (0)	5.26 (1)	0.00 (0)	15.79 (3)
Romaine lettuce + Heart	36	0.00 (0)	0.00 (0)	2.78 (1)	2.78 (1)	5.56 (2)	5.56 (2)	0.00 (0)	16.67 (6)
Cilantro	24	0.00 (0)	0.00 (0)	8.33 (2)	0.00 (0)	0.00 (0)	4.17 (1)	0.00 (0)	12.50 (3)
Mixed lettuce	9	0.00 (0)	0.00 (0)	0.00 (0)	0.00 (0)	0.00 (0)	11.11 (1)	0.00 (0)	11.11 (1)
Carrot	9	0.00 (0)	0.00 (0)	0.00 (0)	0.00 (0)	0.00 (0)	0.00 (0)	0.00 (0)	0.00 (0)
Mixed vegetables	40	0.00 (0)	2.50 (1)	0.00 (0)	0.00 (0)	0.00 (0)	2.50 (1)	0.00 (0)	5.00 (2)
Tomato	54	0.00 (0)	0.00 (0)	3.70 (2)	1.85 (1)	1.85 (1)	0.00 (0)	0.00 (0)	7.41 (4)
EDIBLE FUNGI	22	0.00 (0)	0.00 (0)	9.09 (2)	4.55 (1)	0.00 (0)	0.00 (0)	0.00 (0)	13.64 (3)
Mushrooms	22	0.00 (0)	0.00 (0)	9.09 (2)	4.55 (1)	0.00 (0)	0.00 (0)	0.00 (0)	13.64 (3)
RAW MEAT	121	2.48 (3)	0.00 (0)	1.65 (2)	1.65 (2)	1.65 (2)	2.48 (2)	0.83 (1)	10.74 (13)
Raw Ground Beef	36	2.78 (1)	0.00 (0)	0.00 (0)	3.33 (1)	0.00 (0)	3.33 (1)	2.78 (1)	13.89 (5)
Raw Beef	30	0.00 (0)	0.00 (0)	3.33 (1)	0.00 (0)	0.00 (0)	3.33 (1)	0.00 (0)	6.67 (2)
Raw Chicken	42	2.38 (1)	0.00 (0)	2.38 (1)	2.38 (1)	2.38 (1)	0.00 (0)	0.00 (0)	9.52 (4)
Ground Pork	3	0.00 (0)	0.00 (0)	0.00 (0)	0.00 (0)	0.00 (0)	0.00 (0)	0.00 (0)	0.00 (0)
Raw Pork	10	10.00 (1)	0.00 (0)	0.00 (0)	0.00 (0)	10.00 (1)	0.00 (0)	0.00 (0)	20.00 (2)
UNPROCESSED DAIRY	48	4.17 (2)	2.08 (1)	0.00 (0)	2.08 (1)	0.00 (0)	4.17 (2)	2.08 (1)	14.58(7)
Raw Milk	48	4.17 (2)	2.08 (1)	0.00 (0)	2.08 (1)	0.00 (0)	4.17 (2)	2.08 (1)	14.58(7)
PROCESSED MEAT	39	0.00 (0)	0.00 (0)	0.00 (0)	0.00 (0)	0.00 (0)	0.00 (0)	0.00 (0)	0.00 (0)
Pork Ham	11	0.00 (0)	0.00 (0)	0.00 (0)	0.00 (0)	0.00 (0)	0.00 (0)	0.00 (0)	0.00 (0)
Pork Sausage	9	0.00 (0)	0.00 (0)	0.00 (0)	0.00 (0)	0.00 (0)	0.00 (0)	0.00 (0)	0.00 (0)
Beef Hotdog	16	0.00 (0)	0.00 (0)	0.00 (0)	0.00 (0)	0.00 (0)	0.00 (0)	0.00 (0)	0.00 (0)
Smoked Pork	3	0.00 (0)	0.00 (0)	0.00 (0)	0.00 (0)	0.00 (0)	0.00 (0)	0.00 (0)	0.00 (0)
PROCESSED DAIRY	196	0.00 (0)	0.00 (0)	0.00 (0)	0.00 (0)	0.00 (0)	0.00 (0)	0.50 (1)	0.50 (1)
Whole Milk	25	0.00 (0)	0.00 (0)	0.00 (0)	0.00 (0)	0.00 (0)	0.00 (0)	0.00 (0)	0.00 (0)
Milk (2%)	25	0.00 (0)	0.00 (0)	0.00 (0)	0.00 (0)	0.00 (0)	0.00 (0)	0.00 (0)	0.00 (0)
Mozzarella Cheese	17	0.00 (0)	0.00 (0)	0.00 (0)	0.00 (0)	0.00 (0)	0.00 (0)	0.00 (0)	0.00 (0)
Cream Cheese	14	0.00 (0)	0.00 (0)	0.00 (0)	0.00 (0)	0.00 (0)	0.00 (0)	7.14 (1)	7.14 (1)
Sandwich Cheese	9	0.00 (0)	0.00 (0)	0.00 (0)	0.00 (0)	0.00 (0)	0.00 (0)	0.00 (0)	0.00 (0)
Pepper Jack Cheese	12	0.00 (0)	0.00 (0)	0.00 (0)	0.00 (0)	0.00 (0)	0.00 (0)	0.00 (0)	0.00 (0)
Snack Cheese	3	0.00 (0)	0.00 (0)	0.00 (0)	0.00 (0)	0.00 (0)	0.00 (0)	0.00 (0)	0.00 (0)
Parmesan Cheese	9	0.00 (0)	0.00 (0)	0.00 (0)	0.00 (0)	0.00 (0)	0.00 (0)	0.00 (0)	0.00 (0)
Skimmed Milk Cheese	6	0.00 (0)	0.00 (0)	0.00 (0)	0.00 (0)	0.00 (0)	0.00 (0)	0.00 (0)	0.00 (0)
Cheddar Cheese	12	0.00 (0)	0.00 (0)	0.00 (0)	0.00 (0)	0.00 (0)	0.00 (0)	0.00 (0)	0.00 (0)
Feta Cheese	30	0.00 (0)	0.00 (0)	0.00 (0)	0.00 (0)	0.00 (0)	0.00 (0)	0.00 (0)	0.00 (0)
Yogurt	34	0.00 (0)	0.00 (0)	0.00 (0)	0.00 (0)	0.00 (0)	0.00 (0)	0.00 (0)	0.00 (0)
TOTAL STUDIED POOL	861	0.81 (7)	0.69 (6)	1.85 (16)	1.27 (11)	1.15 (10)	2.08 (18)	0.58 (5)	8.48 (73)

* *L. mono-L. monocytogenes*.

**Table A2:** Taxonomic Distribution, and Public Health Risk Recovered *Listeria* Isolates (N = 74).

Clinical Cohort and Species	Isolation Count (n)	Proportion of Total Genus Incidence (%) (n = 74)	Incidence/Prevalence in Total Retail Samples (N = 866)
Pathogenic Cohort	13	17.57	1.50
*L. monocytogenes*	7	9.46	0.81
*L. ivanovii*	6	8.11	0.69
Non-Pathogenic Cohort	61	82.43	7.04
*L. welshimeri*	18	24.32	2.08
*L. innocua*	16	21.62	1.85
*L. seeligeri*	11	14.86	1.27
*L. grayi*	10	13.51	1.15
Unidentified *Listeria* spp.	6	8.11	0.69
Total Yield	74	100.00	8.55

**Table A3:** Pathogenicity Distribution and Prevalence of *Listeria* Isolates Across Sampled Retail Food Matrices (N = 861).

Food Cohort and Category	Total Sample Size (N)	Pathogenic Cohort ^a^ % (n)	Non-Pathogenic Cohort ^b^ % (n)	Overall Prevalence % (n)
Vegetables	435	1.38% (6)	10.11% (44)	11.49% (49)
Edible Fungi (Mushrooms)	22	0.00% (0)	13.64% (3)	13.64% (3)
Raw Meat	121	2.48% (3)	8.26% (10)	10.74% (13)
Unprocessed Dairy	48	8.33% (4)	6.25% (3)	14.58% (7)
Processed Meat	39	0.00% (0)	0.00% (0)	0.00% (0)
Processed Dairy	196	0.00% (0)	0.50% (1)	0.50% (1)
TOTAL STUDIED	861	1.51% (13)	6.35% (55)	8.55% (73)

^a^ Pathogenic Cohort reflects the combined isolation parameters of *Listeria monocytogenes* (n = 7) and *Listeria ivanovii* (n = 6). ^b^ Non-Pathogenic Cohort reflects the pooled parameters of *Listeria innocua* (n = 16), *Listeria seeligeri* (n = 11), *Listeria grayi* (n = 10), *Listeria welshimeri* (n = 18), and unidentified *Listeria* strains (n = 6).

**Table A4:** Itemized phenotypic antimicrobial resistance, intermediate and susceptibility percentage distributions across individual classical *Listeria* isolates (n = 68).

	*L. monocytogenes* (N = 7) R/I/S % (*N*)	*L. ivanovii* (N = 6) R/I/S % (*N*)	*L. innocua* (N = 16) R/I/S % (*N*)	*L. seeligeri* (N = 11) R/I/S % (*N*)	*L. grayi* (N = 10) R/I/S % (*N*)	*L. welshimeri* (N = 18) R/I/S % (*N*)
Amikacin	0.0 (0)/0.0 (0)/100 (7)	0.0 (0)/16.7 (1)/83.3 (5)	0.0 (0)/0.0 (0)/100 (16)	0.0 (0)/0.0 (0)/100 (11)	0.0 (0)/20.0 (2)/80.0 (8)	0.0 (0)/0.0 (0)/100 (18)
Azithromycin	0.0 (0)/0.0 (0)/100 (7)	0.0 (0)/0.0 (0)/100 (6)	12.5 (2)/6.3 (1)/81.3 (13)	9.1 (1)/18.2 (2)/72.7 (8)	10.0 (1)/20.0 (2)/70.0 (7)	5.6 (1)/22.2 (4)/72.2 (13)
Chloramphenicol	0.0 (0)/14.3 (1)/85.7 (6)	16.7 (1)/50.0 (3)/33.3 (2)	18.8 (3)/18.8 (3)/62.5 (10)	0.0 (0)/0.0 (0)/100 (11)	0.0 (0)/0.0 (0)/100 (10)	5.6 (1)/5.6 (1)/88.9 (16)
Ciprofloxacin	14.3 (1)/0.0 (0)/85.7 (6)	33.3 (2)/16.7 (1)/50.0 (3)	12.5 (2)/25.0 (4)/62.5 (10)	0.0 (0)/0.0 (0)/100 (11)	30.0 (3)/30.0 (3)/40.0 (4)	22.2 (4)/11.1 (2)/66.7 (12)
Clindamycin	28.6 (2)/14.3 (1)/57.1 (4)	16.7 (1)/66.7 (4)/16.7 (1)	18.8 (3)/12.5 (2)/68.8 (11)	0.0 (0)/18.2 (2)/81.8 (9)	40.0 (4)/20.0 (2)/40.0 (4)	0.0 (0)/0.0 (0)/100 (18)
Erythromycin	14.3 (1)/0.0 (0)/85.7 (6)	0.0 (0)/0.0 (0)/100 (6)	0.0 (0)/0.0 (0)/100 (16)	18.2 (2)/27.3 (3)/54.5 (6)	20.0 (2)/20.0 (2)/60.0 (6)	0.0 (0)/0.0 (0)/100 (18)
Kanamycin	14.3 (1)/14.3 (1)/71.4 (5)	50.0 (3)/0.0 (0)/50.0 (3)	25.0 (4)/25.0 (4)/50.0 (8)	27.3 (3)/36.4 (4)/36.4 (4)	30.0 (3)/20.0 (2)/50.0 (5)	16.7 (3)/27.8 (5)/55.6 (10)
Levofloxacin	42.9 (3)/0.0 (0)/57.1 (4)	66.7 (4)/0.0 (0)/33.3 (2)	18.8 (3)/31.3 (5)/50.0 (8)	18.2 (2)/36.4 (4)/45.5 (5)	40.0 (4)/20.0 (2)/40.0 (4)	0.0 (0)/22.2 (4)/77.8 (14)
Moxifloxacin	14.3 (1)/0.0 (0)/85.7 (6)	16.7 (1)/16.7 (1)/66.7 (4)	18.8 (3)/12.5 (2)/68.8 (11)	18.2 (2)/9.1 (1)/72.7 (8)	20.0 (2)/0.0 (0)/80.0 (8)	16.7 (3)/22.2 (4)/61.1 (11)
Nitrofurantoin	42.9 (3)/14.3 (1)/42.9 (3)	83.3 (5)/0.0 (0)/16.7 (1)	37.5 (6)/12.5 (2)/50.0 (8)	36.4 (4)/18.2 (2)/45.5 (5)	60.0 (6)/20.0 (2)/20.0 (2)	44.4 (8)/5.6 (1)/50.0 (9)
Penicillin G	57.1 (4)/0.0 (0)/42.9 (3)	66.7 (4)/0.0 (0)/33.3 (2)	25.0 (4)/0.0 (0)/75.0 (12)	36.4 (4)/18.2 (2)/45.5 (5)	50.0 (5)/10.0 (1)/40.0 (4)	11.1 (2)/5.6 (1)/83.3 (15)
Rifampin	42.9 (3)/0.0 (0)/57.1 (4)	16.7 (1)/0.0 (0)/83.3 (5)	12.5 (2)/0.0 (0)/87.5 (14)	0.0 (0)/0.0 (0)/100 (11)	10.0 (1)/10.0 (1)/80.0 (8)	11.1 (2)/16.7 (3)/72.2 (13)
Tetracycline	0.0 (0)/0.0 (0)/100 (7)	0.0 (0)/0.0 (0)/100 (6)	6.3 (1)/0.0 (0)/93.8 (15)	0.0 (0)/0.0 (0)/100 (11)	0.0 (0)/0.0 (0)/100 (10)	0.0 (0)/0.0 (0)/100 (18)
Tobramycin	0.0 (0)/0.0 (0)/100 (7)	0.0 (0)/0.0 (0)/100 (6)	0.0 (0)/0.0 (0)/100 (16)	0.0 (0)/0.0 (0)/100 (11)	0.0 (0)/0.0 (0)/100 (10)	5.6 (1)/0.0 (0)/94.4 (17)
Trimethoprim	14.3 (1)/0.0 (0)/85.7 (6)	16.7 (1)/0.0 (0)/83.3 (5)	12.5 (2)/6.3 (1)/81.3 (13)	18.2 (2)/0.0 (0)/81.8 (9)	30.0 (3)/30.0 (3)/40.0 (4)	11.1 (2)/0.0 (0)/88.9 (16)
TMP-SMX ^A^	0.0 (0)/0.0 (0)/100 (7)	0.0 (0)/0.0 (0)/100 (6)	0.0 (0)/0.0 (0)/100 (16)	0.0 (0)/0.0 (0)/100 (11)	0.0 (0)/0.0 (0)/100 (10)	5.6 (1)/0.0 (0)/94.4 (17)

^A^ TMP-SMX: Trimethoprim–Sulfamethoxazole. R—Resistant; I—Intermediate; S—Susceptible.
